# Fermentation of Milk in the Mammæ

**Published:** 1861-01

**Authors:** 


FERMENTATION OF MILK IN THE MAMMÆ.—At the last meeting of the British Association for the advancement of science, in the section of Physiolgy, Dr. Gibbs, as appears from the proceedings published in the Lancet, read a paper on the saccharine fermentation within the female breast, and its influence on the child. He showed that from various causes of a constitutional nature, in which the nervous sysstem played an important part, the saccharine element of the milk underwent fermentation at the moment of its secretion, and gave rise to the generation of two species of animalcules, namely, vibriones and monads. The milk containing these was usually rich in sugar, but, owing to the fact of its having undergone fermentation within the gland itself, its healthy character was destroyed, and it was not therefore capable of assimilation within the stomach of the infant, as evidenced by the most extreme degree of emaciation—in fact, the child was undergoing starvation. The animalcules were developed within the breasts. The author had proved the correctness of his views in a series of experiments and researches into this question since 1854. In the discussion which ensued, much credit was given to the author for his labors in this novel field of inquiry; and numerous questions were put to him in relation to the condition of the blood and other fluids, in such conditions as he had described.

